# Supplementary material for: Elevated Soluble Galectin-3 as a Marker of Chemotherapy Efficacy in Breast Cancer Patients: A Prospective Study
Source: Int J Breast Cancer. 2020 Mar 14;2020:4824813. doi: 10.1155/2020/4824813 (PMC7097759; doi:10.1155/2020/4824813)
Supplement: Supplementary Materials — Supplemental Fig. 1: plasma levels of Galectin-3 decrease with increasing grade of tumor in breast cancer patients. Supplemental Fig. 2: plasma Gal-3 levels increase in response to chemotherapy. Supplemental Fig. 3: chemotherapy-induced extracellular Gal-3 correlates with apoptosis in cancer cells. Supplemental Table I: patient characteristics for breast cancer patients receiving adjuvant chemotherapy after surgical removal of primary tumor. Supplemental Table II: patient characteristics for breast cancer patients receiving neoadjuvant chemotherapy followed by surgery. [file 4824813.f1.pdf]

Supplemental Material:

Supplemental Fig. 1:

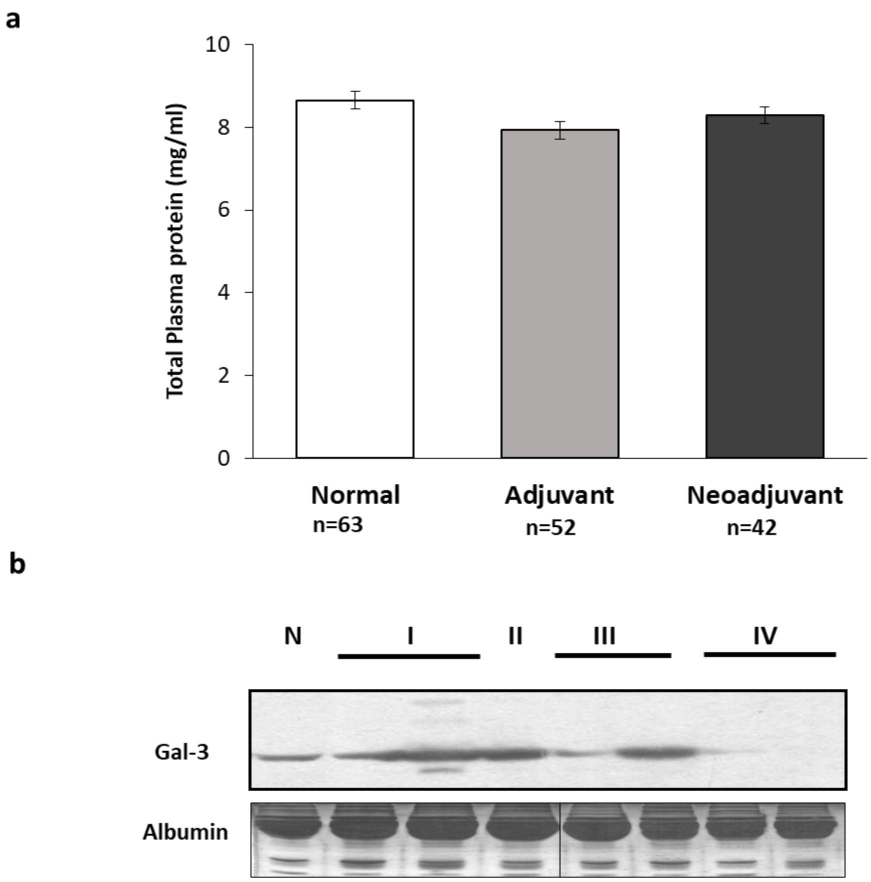

Supplemental Fig. 1: Plasma levels of Galectin-3 decrease with increasing grade of tumor in breast cancer patients. a. Total protein levels in plasma remain unchanged regardless of disease state in cancer patients prior to any treatment. **b.** A representative western blot showing relative Gal-3 expression in plasma from patients with varying grades of breast cancer and non-tumoral control. Albumin band on silver stained gel is used as a loading control.

Supplemental Fig. 2

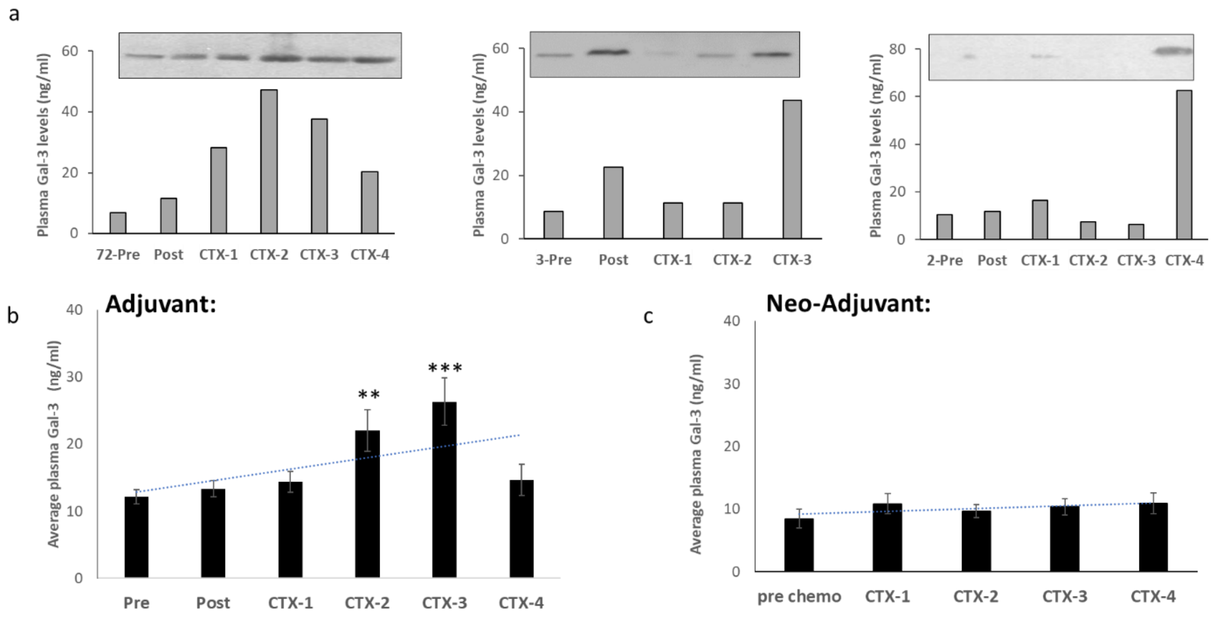

Supplemental Fig. 2: Plasma Gal-3 levels increase in response to chemotherapy. a. Representative ELISA quantification graphs and matched western blots confirming chemotherapy induced rise in plasma Gal-3 levels. Patient study number is listed at the beginning of each graph. Initial levels at the time of diagnosis (Pre), 2-weeks post surgical removal of tumor (Post) and after each chemotherapy cycle (CTX-1 to CTX-4) are shown. **b** Average plasma Gal-3 levels were calculated at the time of diagnosis (pre), 2 weeks post-surgical tumor removal (Post) and after each chemotherapy cycle (CTX-1-4) in patients receiving adjuvant chemotherapy (n=45). Notice significant increase in Gal-3 levels with each chemotherapy cycle in adjuvant patients (Pre:CTX2 p=0.008812; Pre:CTX3 p=0.000717 compared to initial (Pre) levels).

Supplemental Fig. 3

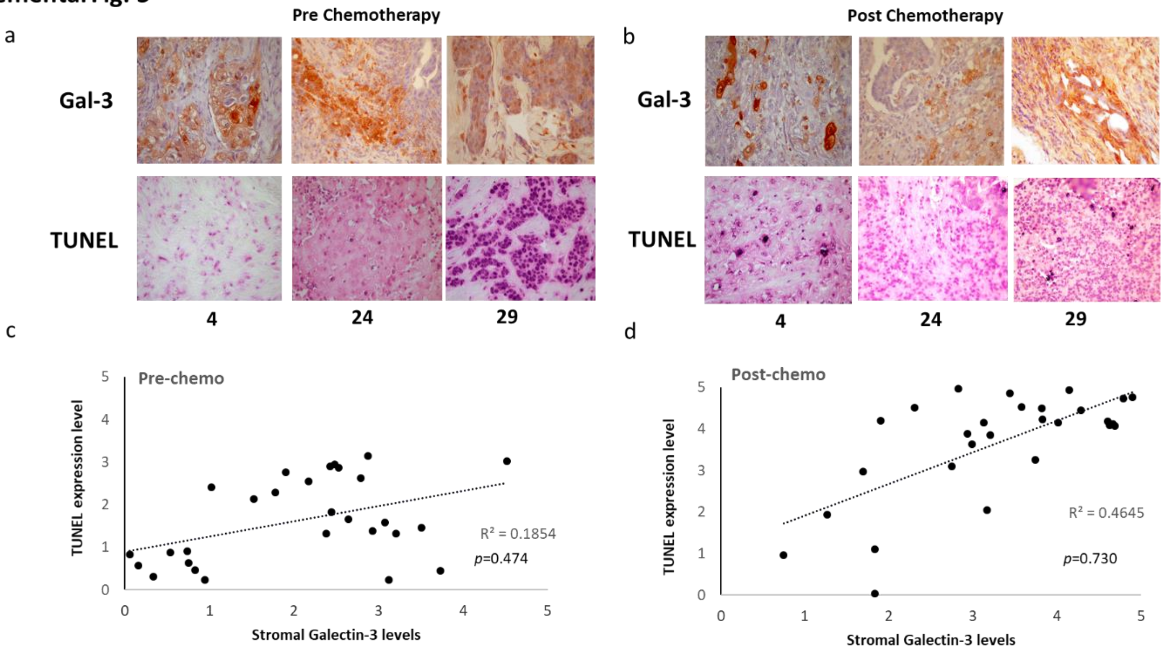

Supplemental Fig. 3: Chemotherapy induced extracellular Gal-3 correlates with apoptosis in cancer cells. a. TUNEL analysis of pre and post chemotherapy tumor tissue indicate positive relationship between stromal Gal-3 expression and apoptosis. Tissues with higher levels of stromal Gal-3 also show increase TUNEL reactivity. **b.** Correlation graph depicting the relationship between stromal Gal-3 expression and TUNEL reactivity. Pearson coefficient calculation was used to examine correlation. There was a strong positive correlation between stromal Gal-3 and TUNEL expressions post-chemotherapy (p=0.730). These results suggest an anti-tumor effect of soluble Gal-3.

Supplemental Table I: Patient characteristics for breast cancer patients receiving Adjuvant chemotherapy after surgical removal of primary tumor. Patient age, tumor size, type, grade and metastatic status are given. TRN=Triple negative; ER= Estrogen receptor; PR= Progesterone receptor; IDC=Intra-ductal carcinoma; DCIS= Ductal carcinoma in-situ; LCA= Lobular carcinoma; AC-T=Doxorubicin hydrochloride (Adriamycin) & Cyclophosphamide followed by Taxol; FAC/T=Fluorouracil, Doxorubicin, Cyclophosphamide followed by Taxol; CMF=Cyclophosphamide, Methotrexate, Fluorouracil.

ADJUVANT Patient Info								
**ID**	**Age**	**Tumor type**	**Grade**	**Ln status**	**ER**	**PR**	**Her2Neu**	**Chemo type**
**1**	41	IDC	III	+	-	-	2+	AC/ T
**2**	29	IDC	III	+	-	-	-	AC/T
**3**	39	IDC	III	-	-	+	-	CMF/T
**6**	34	IDC	II	+	-	-	3+	AC/ T
**7**	43	IDC	II	+	+	+	-	CMF/ T
**8**	53	IDC	II/ III	+	-	-	+	FAC/T
**9**	64	IDC	II	+	+	+	-	FAC/ T
**10**	43	IDC	II	-	+	+	2+	CMF/T
**46**	63	IDC	II	+	+	-	-	FAC/T
**47**	29	IDC	II	+	-	-	-	AC/T
**48**	37	IDC	III	+	-	+	-	AC/T
**49**	43	IDC	II	+	-	-	-	FAC/T
**50**	59	IDC	III	N/A	+	+	+	AC/T
**51**	54	IDC	III	+	-	-	-	AC/T
**52**	32	IDC	II	+	+	+	2+	AC/T
**53**	36	IDC	II	+	-	-	+	AC/T
**54**	44	IDC	III	-	+	+	-	FAC
**55**	44	IDC	II	+	+	+	2+	AC/T
**56**	28	IDC	III	+	-	-	-	AC/T
**57**	38	IDC	III	+	-	-	-	FAC
**58**	59	IDC	III	-	N/A	N/A	N/A	AC/T
**59**	51	IDC	III	+	+	-	2+	FAC/T
**60**	54	IDC	III	+	+	-	-	FAC
**68**	39	IDC	III	+	+	-	3+	AC/T
**71**	37	IDC	II	+	75% +	90% +		Taxol
**72**	49	IDC	II	+	+	+	-	AC/T
**73**	40	IDC	III	+	-	-	-	AC/T
**74**	44	IDC	III	+	+	-	-	FAC/T
**75**	42	IDC	II	+	-	+	1+	AC/T
**76**	39	Sarcoma	III	+	N/A	N/A	N/A	AC/T
**77**	34	IDC	III	-	-	-	-	AC/T
**78**	56	ILC	II	-	N/A	N/A	N/A	AC/T
**79**	54	IDC	III	-	N/A	N/A	N/A	AC/T
**80**	51	IDC	III	-	N/A	N/A	N/A	AC/T
**81**	57	IDC	III	-	-	-	-	AC/T
**82**	46	IDC	II	+	+	+	-	AC/T
**83**	56	ILC	II	+	N/A	N/A	N/A	AC/T
**84**	59	IDC	II	-	+	+	-	AC/T

**85**	64	IDC	II	+	N/A	N/A	N/A	FAC/T
**86**	59	IDC	III	+	+	-	3+	FAC/T
**87**	34	IDC	III	-	+	+	2+	AC/T
**88**	39	IDC	III	-	+	+	-	FAC
**89**	47	ILC	IV	+	-	30% +	-	AC/T
**90**	45	IDC	III	+	<10%	<10%	-	AC/T
**91**	46	IDC	II	N/A	N/A	N/A	N/A	AC.T
**101**	55	IDC	II	-	-	-	3+	AC/T
**102**	44	IDC	III	+	<10%	<10%	-	AC/T
**103**	33	IDC	III	+	-	-	2+	AC/T
**104**	47	IDC	II	+	20-40% +	10-15% +	-	AC/T
**105**	65	IDC/ ILC	II	+	80% +	50% +	-	AC/T
**106**	55	IDC	II	-	10% +	-	2+	FAC/T
**107**	56	IDC	II	+	10%+	10%+	2+	AC/T

TRN=Triple negative; ER= Estrogen receptor; PR= Progesterone receptor; IDC=Intra-ductal carcinoma; DCIS= Ductal carcinoma in-situ; LCA= Lobular carcinoma; AC-T=Doxorubicin hydrochloride (Adriamycin) & Cyclophosphamide followed by Taxol; FAC/T=Fluorouracil, Doxorubicin, Cyclophosphamide followed by Taxol; CMF=Cyclophosphamide, Methotrexate, Fluorouracil.

Supplemental Table II: Patient characteristics for breast cancer patients receiving Neo-adjuvant chemotherapy followed by surgery. Patient age, tumor size, type, grade and metastatic status are given. TRN=Triple negative; ER= Estrogen receptor; PR= Progesterone receptor; IDC=Intra-ductal carcinoma; DCIS= Ductal carcinoma in-situ; LCA= Lobular carcinoma. AC-T=Doxorubicin hydrochloride (Adriamycin) & Cyclophosphamide followed by Taxol; FAC/T=Fluorouracil, Doxorubicin, Cyclophosphamide followed by Taxol; CMF=Cyclophosphamide, Methotrexate, Fluorouracil.

Neo-adjuvant Patient Info								
**ID**	**Age**	**Tumor type**	**Grade**	**Ln status**	**ER**	**PR**	**Her2Neu**	**Chemo type**
**4**	58	IDC	III	-	-	-	-	FAC/T
**5**	35	IDC	III	-	+	+	-	FAC/T
**21**	26	IDC	N/A	-	-	-	2+	AC/T
**22**	48	Metastatic Ca	N/A	+	-	-	-	AC/T
**23**	49	IDC	III	+	-	-	3+	AC/T
**24**	41	IDC	III	+	-	-	3+	FAC/T
**25**	39	IDC	III	+	30%	20%	3+	FAC/T
**26**	39	ILC	II	+	-	-	3+	FAC/T
**27**	52	IDC	III	+	-	-	-	AC/T
**28**	40	IDC	III	+	-	-	-	AC/T
**29**	48	IDC	II	-	60-70	70-80	-	AC/T
**30**	39	IDC	III	-	+	+	-	AC/T
**67**	59	IDC	II	+	80	90	-	AC/T
**69**	47	IDC	I	-	35-40	-	-	Carboplatin/ T
**70**	62	IDC	II	+	50 %	10%	-	AC/T
**94**	44	IDC	II	-	-	-	3+	FAC/T
**98**	54	ILC	II	+	+	+	-	FAC/ T
**99**	44	IDC	III	+	-	-	-	AC/T
**92**	41	LCIS	III	-	+	-	-	AC/T
**93**	47	IDC/ ILC	II	-	5%	5%	-	AC/T
**95**	43	IDC/ ILC	II	-	5%	5%	-	AC/T
**96**	48	IDC	III	+	-	-	-	AC/T
**97**	52	IDC	III	-	-	-	-	AC/T
**100**	20	IDC	III	+	-	-	2+	FAC/ T
**108**	54	IDC	IV	N/A	N/A	N/A	N/A	FAC/ 5FU/ T
**109**	39	IDC	II	-	60%	30%	N/A	AC/ T
**110**	42	IDC	III	+	50%	-	-	AC/ T/ Doc
**111**	55	IDC	III	+	N/A	N/A	N/A	FAC/T
**112**	50	IDC	III	+	-	-	-	AC/ T
**113**	39	IDC	II	-	-	-	+	AC/ T
**114**	30	IDC	III	+	-	-	-	Carboplatin/ AC/T
**115**	45	IDC	II	N/A	-	-	3+	FAC/T
**116**	34	IDC	II	N/A	-	-	-	AC/T
**117**	63	IDC	III	N/A	N/A	N/A	N/A	
**118**	49	IDC	II	-	-	-	-	AC/ T
**119**	45	IDC	III	+	-	-	3+	AC/T
TRN=Triple negative; ER= Estrogen receptor; PR= Progesterone receptor; IDC=Intra-ductal carcinoma; DCIS= Ductal carcinoma in-situ; LCA= Lobular carcinoma; AC-T=Doxorubicin hydrochloride (Adriamycin) & Cyclophosphamide followed by Taxol; FAC/T=Fluorouracil, Doxorubicin, Cyclophosphamide followed by Taxol; CMF=Cyclophosphamide, Methotrexate, Fluorouracil.								
